# Using Group Model Building to Capture the Complex Dynamics of Scaling Up District-Level Surgery in Arusha Region, Tanzania

**DOI:** 10.34172/ijhpm.2020.249

**Published:** 2020-12-13

**Authors:** Henk Broekhuizen, Monic Lansu, Jakub Gajewski, Chiara Pittalis, Martilord Ifeanyichi, Adinan Juma, Paul Marealle, Edward Kataika, Kondo Chilonga, Etiënne Rouwette, Ruairi Brugha, Leon Bijlmakers

**Affiliations:** ^1^Department of Health Evidence, Radboud University Medical Center, Nijmegen, The Netherlands.; ^2^Department of Business Administration, Institute for Management Research, Radboud University, Nijmegen, The Netherlands.; ^3^Institute of Global Surgery, Royal College of Surgeons Ireland, Dublin 2, Ireland.; ^4^Division of Population Health Sciences, Royal College of Surgeons in Ireland, Dublin 2, Ireland.; ^5^East Central and Southern Africa Health Community, Arusha, Tanzania.; ^6^Tanzania Surgical Association, Dar Es Salaam, Tanzania.; ^7^Kilimanjaro Christian Medical Centre, Moshi, Tanzania.

**Keywords:** Surgical Mentoring, District Hospital, Tanzania, Participatory Research, Systems Thinking

## Abstract

**Background:** Scaling up surgery at district hospitals (DHs) is the critical challenge if the Tanzanian national Surgical, Obstetric, and Anesthesia Plan (NSOAP) objectives are to be achieved. Our study aims to address this challenge by taking a dynamic view of surgical scale-up at the district level using a participatory research approach.

**Methods:** A group model building (GMB) workshop was held with 18 professionals from three hospitals in the Arusha region. They built a graphical representation of the local system of surgical services delivery through a facilitated discussion that employed the nominal group technique. This resulted in a causal loop diagram (CLD) from which the participants identified the requirements for scaling-up surgery and the stakeholders who could satisfy these. After the GMB sessions, we identified clusters of related variables using inductive thematic analysis and the main feedback loops driving the model.

**Results:** The CLD consists of 57 variables. These include the 48 variables that were obtained through the nominal group technique and those that participants added later. We identified 6 themes: patient benefits, financing of surgery, cost sharing, staff motivation, communication, and effects on referral hospital. There are 5 self-reinforcing feedback loops: training, learning, meeting demand, revenues, and willingness to work in a good hospital. There are four self-correcting feedback loops or ‘resistors to change:’ recurrent costs, income lost, staff stress, and brain drain.

**Conclusion:** This study provides a systems view on the scaling up of surgery from a district level perspective. Its results enable a critical appraisal of the feasibility of implementing the NSOAP. Our results suggest that policy-makers should be wary of ‘quick fixes’ that have short term gains only. Long term policy that considers the complex dynamics of surgical systems and that allows for periodic evaluation and adaption is needed to scale up surgery in a sustainable manner.

## Background

**Key Messages**

** Implications for policy makers**

- Stakeholder-based research using group modeling building is essential to obtain a dynamic picture of the ‘reality on the ground’ and to inform and legitimize policy initiatives.
- Scaling up surgery at the district level is a complex undertaking. Our results suggest that policy-makers should be wary of ‘quick fixes’ that may increase surgical productivity for a short period only.
- The implementation of the National Surgical, Obstetric, and Anesthesia Plan (NSOAP) in Tanzania requires further investigation into the feasibility of surgical supervision, the issue of staff retention at the district, and the impact of exemptions on the financial sustainability of hospitals.

** Implications for the public**

Most people in Tanzania live in rural areas. Often they lack access to basic surgical services at their local district hospital (DH). The Tanzanian government has made a plan to do more surgery at the DH. We conducted a workshop on scaling up surgery in DHs with 18 health professionals in Arusha, a city in northern Tanzania. We found that it is very complex. Simple solutions may not be sufficient and have unintended consequences now or later. Our main recommendations for meeting the objectives in the national plan are as follows. First, policy-makers need to strengthen local surgical capacity so that unnecessary referrals can be avoided. Second, it is important to retain skilled staff in remote rural settings. Third, due to the country’s exemption policy hospitals lose money on every surgical case they do. A reimbursement scheme is needed to make sure DHs are not ‘punished’ for doing more surgery.

In Tanzania, 19.3% of deaths and 17% of disability-adjusted life years are attributable to diseases amenable to surgery.^1^ To improve access to surgery, the Tanzanian Ministry of Health, Community Development, Gender, Elderly and Children and the President’s Office for Regional Administration and Local Government have formulated a National Surgical, Obstetric, and Anesthesia Plan (NSOAP) in 2018.^2^ It lays out a strategy to overcome specific health system challenges and achieve the government’s target of providing adequate surgical services for all Tanzanians by 2025. As two-thirds of the population live in rural areas, scaling up safe surgery at district hospitals (DHs) is one of the challenges that needs to be overcome to achieve this target.

 Developing realistic and cost-effective plans for scaling-up surgery at the district level requires evidence on resource availability and clarity around expected outcomes. Several approaches can be used to support this planning process. For example, one can conduct cost analyses to quantify the financial implications of infrastructural/staff investments required for surgical scale-up.^3^ The main disadvantage of this is that such analyses are labor intensive. Also, they often employ a cross-sectional design that makes it difficult to extrapolate costs at the current level of surgical service production to future costs in scenarios of higher surgical output levels. But, more importantly, costing studies consider mostly administratively measurable resources while ignoring intangible, non-quantifiable factors that may play a role in whether, and how, scale-up can be realized. For example, staff workload, motivation, and satisfaction are not captured in routine administrative data, yet they are crucial for success.

 Another approach, which is the focus of this paper, is to take a bottom-up perspective and elicit such intangible important factors from stakeholders.^4,5^ It yields a multitude of factors that may affect or result from scale-up. Through a stakeholder-based approach a more comprehensive view can be obtained of the real requirements for scale-up. A major disadvantage, however, is that often the identified requirements are (implicitly) assumed to be independent of each other. In reality, many of the requirements are interdependent and affect one another in unintended ways: surgical systems, like any health service delivery system, are complex and adaptive.^6^ Complex adaptive systems are characterized by feedback loops (eg, the vicious cycle between workload and staff dropout, each strengthening the other) and delays between cause and effect (eg, it takes time to train new staff). All of this makes it hard to forecast how a complex adaptive system will react to a planned intervention or an (unplanned) external influence. There are many examples of well-intended policies not being effective, or sometimes having unintended consequences because dynamic complexity is not taken into account.^7^

 To overcome these weaknesses our study aims to take a dynamic view of surgical scale-up at the district level using a participatory research approach. This approach is called group model building (GMB), designed to increase the understanding of how complex adaptive systems work and which interventions are likely to be most effective and sustainable.^8,9^ The study was conducted as part of the SURG-Africa project, a 4 year intervention trial in 3 countries (Malawi, Tanzania, and Zambia) that supports mentoring of district-level surgical teams by specialists from referral hospitals.^10^

## Methods

###  Data Collection

 A GMB workshop was held in Arusha town in May 2019. The group included 18 health professionals (out of 20 invited) from 3 hospitals in the Arusha region: Mt. Meru regional referral hospital, Meru DH, and Oltrumet DH. Mt. Meru is the referral hospital to which both the Meru and Oltrumet DHs refer surgical patients. Participants comprised a variety of backgrounds (sometimes overlapping): 2 medical officers-in-charge, 2 specialists, 3 (assistant) medical doctors, 2 matrons, 2 nurses, 1 theatre nurse in-charge, 1 driver, 1 procurement officer, 3 hospital secretaries, and 3 accountants. The Regional Medical Officer of Arusha region also participated. The aim of this particular GMB was to build a graphical qualitative representation of the system, working collaboratively with knowledgeable stakeholders from different parts of the system. Therefore, participant selection was done purposively, with a view to include a wide range of perspectives. The resulting causal model thus reflects the group’s combined knowledge about a problem.^11^

 Before starting the interactive part of the workshop, the purpose of the workshop was briefly introduced and participants were encouraged to actively participate and be open to each other’s perspectives and experiences. Guided by 2 facilitators (LB and HB), the participants developed a causal loop diagram (CLD), depicting all the factors that surround the provision of district-level surgery and its possible scale-up, with arrows indicating causal relations. The diagram took shape based on the participants’ contributions and factors were included only with their explicit consent and consensus in the group. We used Vensim (Ventana Systems) system dynamics software to construct the model, which was projected on a screen so all participants could immediately see their contributions to the CLD.

 We selected Meru DH as the case, with the aim to examine its delivery of acute and life-saving surgery, in line with the remit of district level hospitals in Tanzania. Meru DH was chosen over Oltrumet DH because the group considered it more representative of other DHs in Tanzania: Oltrumet has very bad road access and it has a non-standard financial situation (2 DHs in one district). The central factor of interest was: “*Volume of life saving and essential surgery at Meru DH*.”

 Model development consisted of 2 rounds of variable elicitation, using the nominal group technique to build the CLD in a participatory manner.^12^ The nominal group technique was employed as follows. In the first elicitation round, participants considered the prerequisites for and factors determining surgical scale-up at Meru DH, allowing ample room for participants to discuss among themselves. The question they were asked to think about was: “What is required to achieve an increase in the volume of life saving and essential surgery at Meru DH?” Each participant wrote down one or more prerequisites on cards, which one of the facilitators then collected and put up on a whiteboard, identifying duplicates and grouping similar pre-requisites (variables). During this process, we asked participants to explain their reasoning. Once consensus was reached on a particular variable, it was included in the CLD, and arrows indicating possible causal relations were inserted, where appropriate. The second round of variable elicitation followed a similar procedure. This time, the group was asked to think about the question “What would be the consequences of an increase in the volume of life saving and essential surgery at Meru DH?” Again, the model was changed based on the following discussion. After completing the 2 rounds of variable elicitation, the participants were invited to identify any missing variables and discuss the linkages. They then indicated which variables in the CLD they considered most important for scaling-up surgery, and which stakeholders could influence those variables. Finally, the group took a step back from the model building process and reflected on the policy requirements for making the scale-up of district-level surgery a reality, as well as on the generalizability of the CLD to other districts in Tanzania.

###  Data Analysis

 After the GMB sessions, the resulting model was reorganized to improve readability, for example by minimizing the number of crossing arrows or overlapping variable names. We identified clusters of related variables in the CLD using inductive thematic analysis. Furthermore, we positioned variables such that related variables were all close together. We then identified the feedback loops in the CLD and categorized them into self-reinforcing and self-correcting feedback loops. We gave names to the feedback loops based on the variables that they passed through. To improve clarity, we produced a truncated second CLD where we removed all variables that are not part of at least one feedback loop. The cleaned and truncated versions of the CLD were communicated to the participants for validation along with a one page narrative describing the model.

## Results

###  Variable Elicitation

 The variable elicitation for surgical scale-up prerequisites yielded 31 variables, divided over 12 categories (Table 1). The most commonly mentioned category of prerequisites was equipment (n = 9), followed by skills (n = 6) and infrastructure (n = 5). Prerequisites that were only mentioned once were: surgeon, motivation, supplies, transport, wards, communication, and mentoring and monitoring. The second variable elicitation-round yielded 17 potential consequences of scaling up surgery, divided over twelve categories (Table 2). The most commonly mentioned consequences were ‘increased running/maintenance costs’ (n = 3) and ‘over-utilization/scarcity of resources’ (n = 3), followed by ‘increased surgical skills’ (n = 2). Consequences that were mentioned only once were: improved surgical services, increased revenues, workload, decreased referrals out, increased productivity, shortage operating theatre space, more efficiency of work, patient lives saved, and reduced waiting lists.

**Table 1:** Prerequisites for Scaling Up District-Level Surgery, Mentioned More Than Once by Participants During the First Nominal Group Technique

**Category **	**Times Mentioned **
Equipment	9
Skills	6
Infrastructure	5
Human resources for health	2
Affordable costs/financing	2
Other	9
Total	31

**Table 2:** Perceived Consequences of Scaling up DH-Level Surgery Mentioned During the Nominal Group Technique That Were Mentioned More Than Once

**Category **	**Times Mentioned **
*Increased* running/maintenance costs	3
Over-utilization/scarcity of resources	3
*Increased* experience/skills	2

Abbreviation: DH, district hospital. With italics we denote where participants explicitly included language about direction of change.

###  Diagram Building Process and Description of the Final Causal Loop Diagram

**Figure 1:**
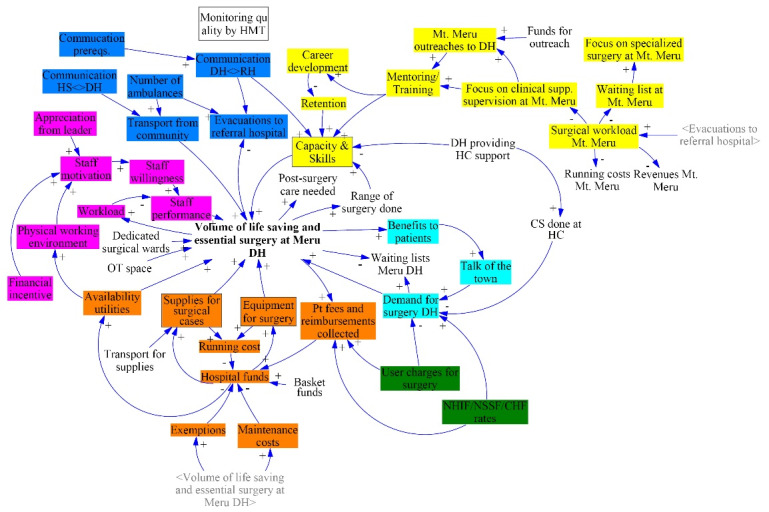
Final CLD. The central variable is in bold in the center. The colors of variables denote their cluster, which we describe in the text of the article. Grey variables are references to other existing variables to make the diagram easier to read. The ‘monitoring quality by HMT’ variable connects to all other variables and these links have been omitted for clarity. Abbreviations: HMT, hospital management team; DH, district hospital; RH, regional hospital; HC, health center; OT, operating theatre; NHIF, national health insurance fund; NSSF, national social security fund; CHF, community health fund; CLD, causal loop diagram.

 While assigning the elicited variables to their places in the CLD, the group explored in detail the types of equipment required for surgery and types of personnel whose surgical skills they considered important. These were added as new variables. During the lunch break, the facilitators grouped these particular variables into 3 categories: ‘supplies for surgery,’ ‘equipment for surgery,’ and ‘capacity and skills;’ where the latter was understood by the group to refer to skills of surgical team members. The group also expanded on other variables in the discussion, such as ‘financing,’ by adding variables that they had overlooked earlier. The CLD that resulted from the group discussion is presented in Figure 1. It consists of 57 variables. These include the 48 that were obtained through the nominal group technique and those that the group added during the discussion, for example for clarification. The CLD has 19 feedback loops affecting the central variable. Each one of the 6 identified themes comprising a set of interconnected variables, as indicated with colors in Figure 1.

 The most important theme (in terms of number of variables) concerns the financial affordability and long term sustainability of scale-up: the orange variables in Figure 1. On the revenue side, an increase in the volume of surgery implies more income from patient fees and insurance reimbursements. On the costs side, scale-up entails more (intensive) use of supplies and equipment, leading to higher procurement and running costs. Another consequence is that with a higher volume of surgery, there will be more patient fee exemptions. In Tanzania, several subgroups of patients (eg, children below 5 years of age, people above 60 years of age, pregnant women, and prisoners) are exempted from paying user fees. To compensate for revenues foregone due to exemptions, participants indicated that hospitals can increase patient fees or negotiate higher health insurance reimbursement rates (green variables in Figure 1). In a related theme, the sustainability of a surgical scale-up involves not only the balance of costs versus revenues, but also on staff factors such as motivation (pink variables in Figure 1). With increased volume of surgery the workload for staff would increase, possibly leading to stress and reduced performance. Participants suggested that this could to some extent be counteracted by financial incentives, appreciation from their supervisors, and a conducive working environment.

 Another important theme in the CLD relates to factors outside of the DH, notably communications and surgical referrals (dark blue and yellow variables in Figure 1). Good communication and ambulances are required for referrals. There are 2 types of referrals for a DH: incoming and outgoing. Incoming referred cases come from lower level facilities in the referral chain. Such referrals increase the surgical workload at the DH. Similarly, outgoing referrals from the DH affect its upstream hospital, Mt. Meru regional hospital. If DH staff can handle more cases locally, referrals to Mt. Meru would decrease, and conversely a reduced surgical capacity at the DH would lead to more outgoing referrals. Mt. Meru would benefit from fewer surgical referrals, as it currently has a long surgical waiting list because of limited operating theatre space. Elective cases are often put on a waiting list or are sent home when emergency cases present, which receive priority. If the burden of simpler elective surgical cases could be treated elsewhere, ie, at district level hospitals, Mt. Meru Regional Hospital could focus more on specialized surgery; and free up surgical specialists to supervise district surgical teams, increasing their surgical skills and potentially leading to more district-level surgery. As a final theme the group mentioned that when more surgery is done at the DH, this could entice patients to come to the hospital who are now staying home with treatable conditions (light blue variables).

**Figure 2:**
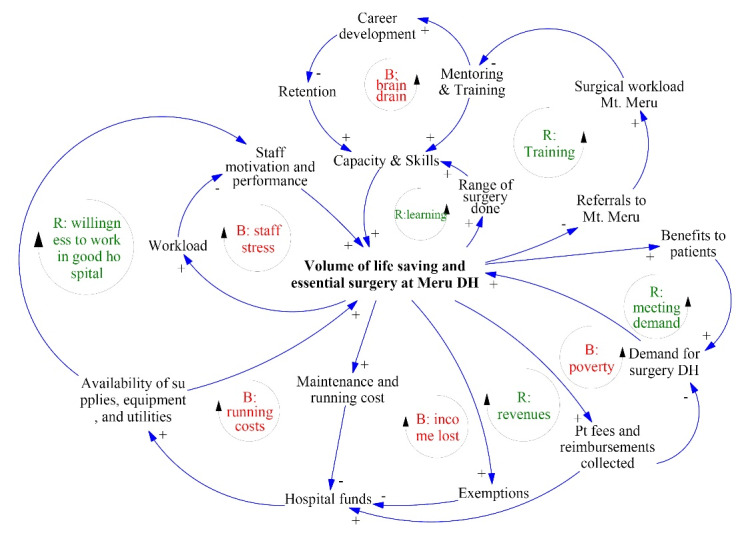
Main Feedback Loops in the Model. Note that many variables that were not in feedback loops were removed, and that some were combined for clarity of presentation. The color of the feedback loops denotes if they are beneficial (green) or harmful (red). Furthermore, B denotes a balancing (self-correcting) and R denotes a reinforcing feedback loop. Abbreviation: DH, district hospital.

 Complex adaptive systems, like the surgical system under study here, are characterized by feedback loops. These are chains of causal relations that form a cycle or a loop. How a system responds to an intervention is mainly determined by the feedback loops it contains. The main feedback loops are presented in Figure 2. There are 5 self-reinforcing feedback loops, namely: training, learning, meeting demand, revenues, and willingness to work in a good hospital. There are 5 self-correcting feedback loops or ‘resistors to change:’ running costs, income lost, staff stress, poverty, and brain drain. The self-reinforcing loops in our model are beneficial for scale-up because they will become stronger over time. In other words: the effects of learning, meeting demand, revenues, and willingness to work in a high performing hospital will all reinforce themselves and become larger. The self-correcting feedback loops have undesired effects as they tend to ‘pull’ the DH back to its initial situation, before the scale-up. For example, the running costs and the exemptions will increase if more surgery is performed, making it hard for the DH to sustain scale-up. Similarly, staff stress and brain drain are both effects that over time tend to undo any surgical scale-up.

###  Generalizability and Levers for Change

**Figure 3:**
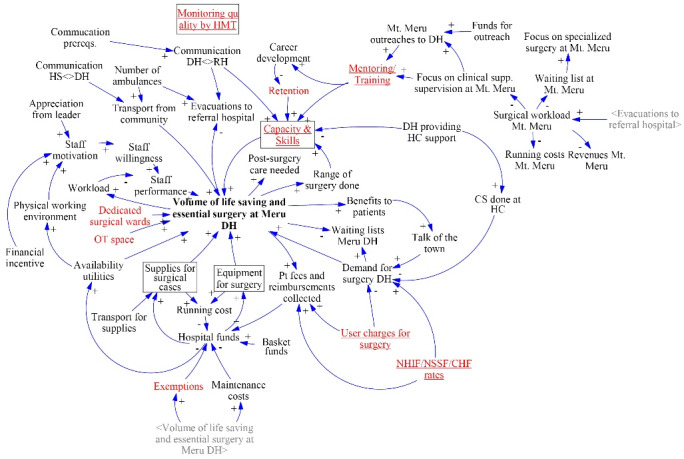
Levers for Change Denoted in Red as Identified by the Participants. Variables with underlined text are those levers for change that the participants felt they could influence to some extent. Grey variables are references to other existing variables to make the diagram easier to read. The ‘monitoring quality by HMT’ variable connects to all other variables and these links have been omitted for clarity. Abbreviations: HMT, hospital management team; DH, district hospital; RH, regional hospital; HC, health center; OT, operating theatre; NHIF, national health insurance fund; NSSF, national social security fund; CHF, community health fund.

 After completing the CLD the participants discussed the model’s generalizability, reckoning that it would apply to other district level hospitals in Tanzania. They were then asked to consider which variables had the most impact in their opinion and which of those hospital management teams (HMTs) could influence (Figure 3). Four variables were selected as important although they are not under HMT influence: having dedicated surgical wards, sufficiency of operating theatre space, staff retention, and fee exemptions. The first 2 are infrastructural requirements. In the Tanzanian health system these fall under central government control. Staff retention of all members of the surgical team was considered important because if these are not retained at the district level, it may undo the effects of mentoring and training. However, participants found staff turnover hard to alter because it seems reasonable for certain staff to develop their careers outside of the district. Fee exemptions were mentioned as important because of their major impact on the financial sustainability of surgery. Participants indicated that the income foregone due to exemptions can be of the same order of magnitude as the total revenues collected by the hospital. Other variables considered important and within the sphere of influence of DH or regional hospital staff, at least to some extent, included quality of care monitoring by HMT, mentoring/training, capacity & skills, user charges for surgery, and insurance reimbursement rates. Monitoring was seen as affecting almost everything else in the CLD; it could contribute significantly to a better understanding of relevant issues. Mentoring/training and capacity/skills were also variables that the group considered they had control over, especially the surgically active participants. Lastly, 2 financial variables were identified that HMT or the regional medical officer have some control over: patient fees and insurance reimbursement rates. If an increase in these could be negotiated it would positively affect the revenue/cost balance. Some participants, however, expressed their ethical concerns that this could exclude poor patients who would not show up at the hospital for surgery if the fees or exemption barriers are too high.

## Discussion

 In this study, we examined the dynamics around the scale-up of surgery at the district level from the perspective of local stakeholders in the Arusha region of Tanzania. The group mapped prerequisites and consequences of surgical scale-up at the district level. The study took a holistic systems view and identified several factors and feedback loops in the system that may be relevant when considering the Tanzanian NSOAP that is currently progressing towards implementation.^4,13^

- The NSOAP articulates that outreach services from zonal and regional hospitals to lower level facilities must be strengthened. We agree and would like to add that it is important that such a service takes the form of a mentoring/supervision program so that there can be a transfer of skills and knowledge to the DH, rather than focus on surgical outreach ‘camps’ where specialist surgeons deliver surgery themselves. If there is no transfer of knowledge and skills, any gains made during outreach are likely to be temporary. In terms of the CLD, policy should take a long-term perspective and invest in strengthening local surgical capacity reinforcing feedback loops.
- The majority of major surgical procedures in Tanzania are performed by non-physician clinicians (NPCs): 85% of caesarean sections^14^ and 71% of non-obstetric procedures.^15^ Their retention, therefore, is critical.^15^ Several studies demonstrate that sustainable district surgical services in many African countries are dependent on the retention of surgically trained NPCs.^16-18^ Hence, policies aimed at improving capacity at the DH (namely, task shifting and training) may be self-defeating in the long term if these workers cannot be retained. There is a lot of emphasis in the NSOAP on staff training, but employment conditions need be sufficiently attractive as well. This means sufficient positions, employment contracts and adequate salaries for surgical staff at all levels – surgeons and anaesthesiologists, as well as NPCs – and investments in surgical infrastructure at all levels, if retention of staff is to be sustained.
- The results from our study very much echo the recommendations the NSOAP makes in the domain of information management and technology. Our study explored the surgical system from the perspective of district-level stakeholders. As the national surgical system in Tanzania is even more complex than what we have described, it is critical to have monitoring systems in place to anticipate and adapt to effects of policy adjustments. However, not everything that determines the effects of policy and strategy implementation can be readily forecasted, let alone measured. Therefore, we would recommend stronger stakeholder involvement in designing policy based on NSOAP objectives.
- Our findings contrast with some of the literature surrounding the NSOAP, which suggests that “*Increased service delivery may result in increased utilization of SOA services and therefore increased revenue from user fees which can support hospital activities.*”^13^ Our study suggests that this is only part of the picture: increased utilization will also increase the financial burden due to fee exemptions. These exemptions can avert or reduce catastrophic household expenditure for surgery, but they constitute a financial burden for DHs. This could discourage hospitals from actually scaling up surgery. It was not the aim of the workshop to determine the exact balance between increased costs and increased revenues resulting from surgical scale-up. However, the group believed (and this is also suggested in economic research we conducted in the region) that an increase in surgery at the DH would increase costs more than it would increase revenues. It is therefore important that this issue be further explored so solutions may be formulated. One approach to reduce the adverse impact of exemptions on hospitals’ financial situation would be to increase health insurance coverage, although this poses challenges in Tanzania where few people have formal employment. Another approach would be to compensate hospitals for income lost due to exemptions.

Much of what is known about barriers to scaling-up district level surgery in sub-Saharan Africa is reflected in our findings, such as the importance of infrastructure, motivated staff, adequate supplies and the financial situation of DHs.^19,20^ When looking specifically at Tanzania, an earlier review by Nyberger et alfound 135 publications about the state of surgery and anesthesia in Tanzania.^4^ Like us, they find that major problems were workforce shortages, inefficiencies in the referral system, lack of water/electricity, and inadequate surgical necessities such as equipment and supplies. Financial and costing studies are scarce, and we found no papers about the impact of user fee exemptions on the financial sustainability of DHs. Sustainability features heavily in growing calls for applying a systems view on surgical policy development in low- and middle-income countries.^21-23^ Systems science poses that all parts of complex adaptive systems are tightly interconnected and that feedback loops determine how they respond to policy intervention.^7^ Policy-makers would need to take interconnectedness and feedback loops into account to maximize health system performance and minimize the risk of unintended consequences of any policy changes. Most publications in Nyberger and colleagues’ review, however, focus on textual descriptions of barriers to surgery without an explicit consideration of interdependencies and feedback loops. This makes developing policy from a systems perspective more difficult. In the present study we have explored such interdependencies and feedback loops for the surgical system in Arusha region. Connected factors in the CLD form several virtuous cycles (training, learning, meeting demand, revenues, and staff satisfaction) that start small but have a tendency to grow over time. However, there are also several self-correcting, sometimes harmful feedback loops (staff stress, running costs, lost hospital income, and brain drain) that have the tendency to undo improvements or aggravate certain negative tendencies. In the field of system science, this combination of feedback loops is a well-known system archetype called ‘limits to growth.’^24^ A key insight from systems science about intervening in such a system is that merely investing in the self-reinforcing feedback loop would be self-defeating: it may improve outcomes in the short term, but these same outcomes will make the self-correcting feedback loops pull the system back to its original state with even greater momentum. In addition to fostering beneficial self-reinforcing feedback loops, policy-makers should try to limit or break the self-correcting feedback loops. Translating this to our case, investing only in staff training and mentoring may produce just a short-term increase in the volume of surgery. Unless issues surrounding workload and financial sustainability are also addressed, DHs will not be able to sustain their higher surgical output.

 On the spectrum of stakeholder inclusion,^25^ GMB is an approach that has been applied successfully in a variety of contexts both to help structure problems, especially those that are complex and dynamic.^26^ Our study supports the notion that GMB is a practical approach that could form part of the toolbox of implementation researchers in their quest to assist policy-makers aiming for surgical policy based on systems science. It is a way to include and combine the views of stakeholders across the healthcare system. This has the benefit of a wider perspective on the system and may help increase the support base for policy changes. In addition, CLDs may include factors not routinely measured in conventional studies and can also be used to put other evidence into context. Furthermore, communicating a message about complexity with a visually attractive CLD derived from stakeholder input may be more effective than using just text or graphs.

 Whether all of these benefits can be realized depends on the uptake and understanding of GMB by the workshop participants. Reflecting on the Arusha workshop we would argue that the method and the underlying systems thinking were quickly adopted by the participants, as primarily indicated by their active involvement in the discussions. Participants were keen to share their views and experiences and they were not shy in discussing different perspectives brought in by others, who sometimes occupied positions higher in the hierarchy. A more specific sign of the group’s adoption of the method was how their wording of variables changed as the workshop progressed. In the first round, many variables needed clarification or did not have a direction (eg, ‘high costs’ versus ‘costs’) and thus did not directly fit into the CLD. In the second round, however, many of the statements on cards included words like ‘increased’ or ‘more,’ indicating the group was starting to grasp the type of reasoning and dynamic features of a CLD. Other signs that indicated the group’s adoption of a systems view were that they started discussing the model in terms of multi-causal relations and feedback loops instead of linear cause-and-effect relations. Nevertheless, there were also some signs of a partial understanding and ownership of the method. Some variables mentioned in the second round were the same as those mentioned in the previous round that asked for prerequisites for surgery, eg, ‘scarcity of resources;’ and some variables were merely re-wordings of the central variable ‘Volume of life saving and essential surgery,’ eg, ‘increased productivity.’

 That the GMB workshop was restricted to one geographic region of Tanzania and involved just 18 participants was its primary limitation. This means that the CLD cannot be taken as a comprehensive reflection of the dynamics surrounding the provision of surgery across all of Tanzania. Rather, it should be seen as the participants’ *dynamic hypothesis* of the phenomena involved in surgical scale-up in their region. This particular CLD focused mostly on the DH context and the only surgical discipline represented was general surgery. This may have led to an overestimation of the beneficial effects of reduced referrals to the hospital as there can be competition between disciplines. Another limitation that may have skewed responses is the fact that some participants were familiar with members of the SURG-Africa research team through previous field work. Although this may have helped them to freely express their opinions and views, they may also have been inclined to give desirable answers (ie, social desirability bias). We have tried to minimize this bias by using the nominal group technique by which participants were given time to reflect and write down their contributions before engaging in a group discussion.

 Given the time constraints and the limited geographical scope of the study, it would not have been realistic to develop concrete policies for surgical scale-up from only the CLD presented in this paper. Although our findings likely capture the most important factors and feedback loops in the context under study, further exploration and subsequent quantitative analyses would be needed in order to inform policy options. The results of this particular study will be used by the SURG-Africa project team in their interactions with national-level stakeholders concerning the sustainability of surgical mentoring alongside results from studies on the costs of performing surgery, surgical referrals, and surgical capacity.

 In order to obtain a more complete picture of the opportunities and obstacles to scale-up so as to inform policy development, one would need to include the perspectives of more stakeholders: eg, national-level stakeholders, health center staff, and patients. It would be beneficial to conduct multiple GMBs across the country and to compare and collate findings. This would allow more comprehensive problem scoping, identification of areas where more research is needed, and trust building among relevant stakeholders. Based on these GMBs, evidence could then be collected so that quantitative systems models can be constructed. Quantitative models are of particular use because they can used for an in-depth exploration of the likely (side) effects of policy options over time.^27^

## Conclusion

 To the best of our knowledge this study is the first to investigate the dynamics of surgical scale-up using GMB. Together with local stakeholders we have explored dynamic issues surrounding scale-up of surgery in the Arusha region of Tanzania. The employed method allows for a systems view of the relevant factors in the local context. Such stakeholder-based research is essential to obtain a picture ‘of reality on the ground’ and to inform and legitimize policy initiatives.^28^ The CLD that the group produced in this study provides local stakeholders with a better insight into the factors that they themselves or their HMTs can influence. At a national level, such a diagram may be a useful piece of information for policy-makers who are planning to scale-up of surgery; and, in the case of Tanzania, to look critically at the feasibility of implementing the NSOAP. Scaling up surgery is a complex undertaking: policy measures that are limited to providing extra inputs would overlook the long term financial consequences and (possibly harmful) unintended side-effects in other domains.^29^ Our results suggest that policy-makers should be wary of ‘quick fixes’ that may increase surgical productivity for a short period only. Long-term policy that considers the complex dynamics of the system and that allows for periodic evaluation and adaption is needed to scale up surgery in a sustainable manner.

## Acknowledgements

 We would like to sincerely thank the participants in the group model building workshop for their time and valuable input. This study was part of the SURG-Africa project, which is funded by the European Union’s Horizon 2020 Programme for Research and Innovation, under grant agreement no: 733391.

## Ethical issues

 This study was approved by the Kilimanjaro Christian Medical College Research Ethics and Review Committee (approval no. CRERC 2026) and the National Institute for Medical Research in Tanzania (approval no. NIMR/HQ/R.8a/Vol. IX/2600).

## Competing interests

 Authors declare that they have no competing interests.

## Authors’ contributions

 HB, ML, and LB were responsible for the conception and design of the study. HB, AJ, EK, and LB partook in the acquisition of data. The analysis and interpretation of the data, as well as the drafting of the manuscript was done by HV, MI, and LB. All co-authors were involved in the critical revision of the manuscript for important intellectual content. Responsible for obtaining funding where JG, PM, EK, RB, and LB. AJ was responsible for administrative support and author LB was responsible for supervision.
